# Supplementary material for: Involvement of cigarette smoke-induced epithelial cell ferroptosis in COPD pathogenesis
Source: Nat Commun. 2019 Jul 17;10:3145. doi: 10.1038/s41467-019-10991-7 (PMC6637122; doi:10.1038/s41467-019-10991-7)
Supplement: Supplementary file 1 — Supplementary information [file 41467_2019_10991_MOESM1_ESM.pdf]

Supplementary Information

Involvement of cigarette smoke-induced epithelial cell ferroptosis in COPD pathogenesis.

Yoshida et al.

This file includes:

Supplementary Figure 1-10

Supplementary Table 1

Supplementary Figure 1.

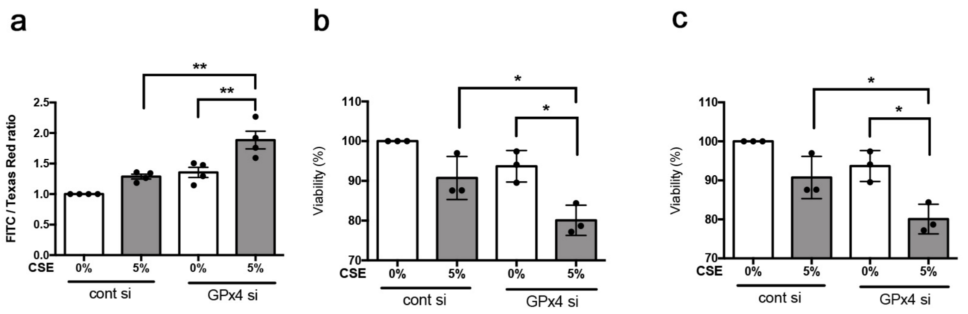

Supplementary Figure 1

CS-induced cell death and lipid peroxidation in alveolar epithelial cells (A549) are regulated by GPx4 knockdown. A549 cells were treated with saline or 5% CSE for 24h and were transfected with control siRNA or GPx4 siRNA 24h before control or 5% CSE treatment. **a.** Lipid peroxidation is assessed by BODIPY 581/591 staining in A549 cells. (n=4 in each group) **b.** LDH assay in A549 cells are shown. (n=3 in each group) **c.** MTT assay in A549 cells are shown. (n=3 in each group) Throughout, data shown represent the mean ±SEM (n=4 per each group). *P<0.05, **P<0.01 by one-way ANOVA followed by Tukey's multiple comparisons test.

Supplementary Figure 2.

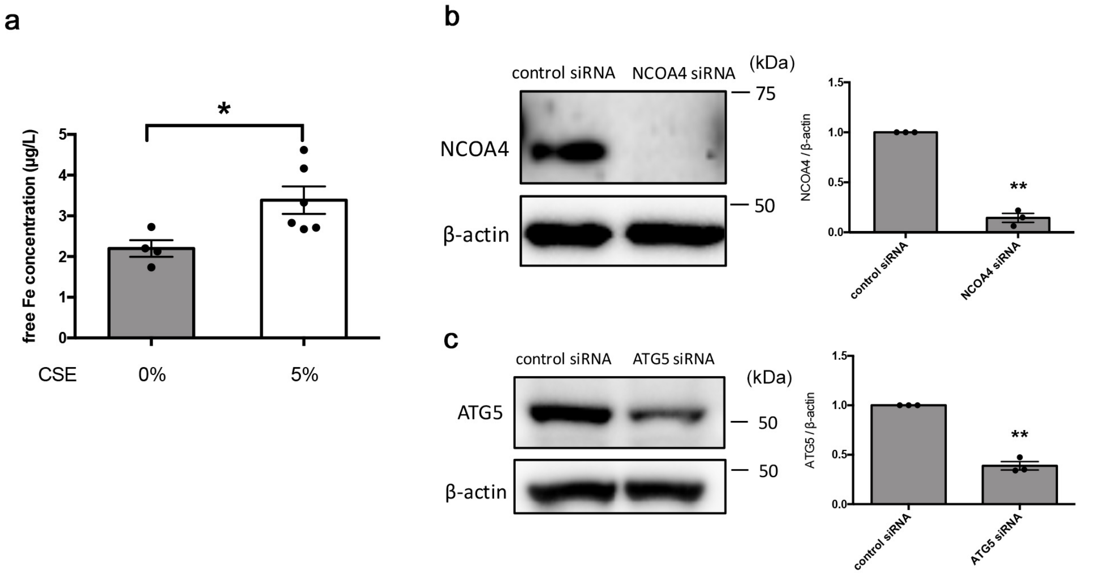

Supplementary Figure 2

a. BEAS-2B cells were treated with 5% CSE for 24 hours. Free iron was measured using ICP-MS in control or 5% CSE treated BEAS-2B. Data shown represent the mean ± SEM (control n=4, CSE n=6). **b.** WB using anti-NCOA4 and anti-β-actin of cell lysates from control siRNA transfected and NCOA4 siRNA transfected BEAS2B.(n=3 in each group) Data shown represent the mean ±SD of NCOA4/ β-actin ratio. **c.** WB using anti-ATG5 and anti-β-actin of cell lysates from control siRNA transfected- and ATG5 siRNA transfected BEAS2B. (n=3 in each group) Data shown represent the mean ±SD of ATG5/ β-actin ratio. **P<0.01 by student t test.

Supplementary Figure 3.

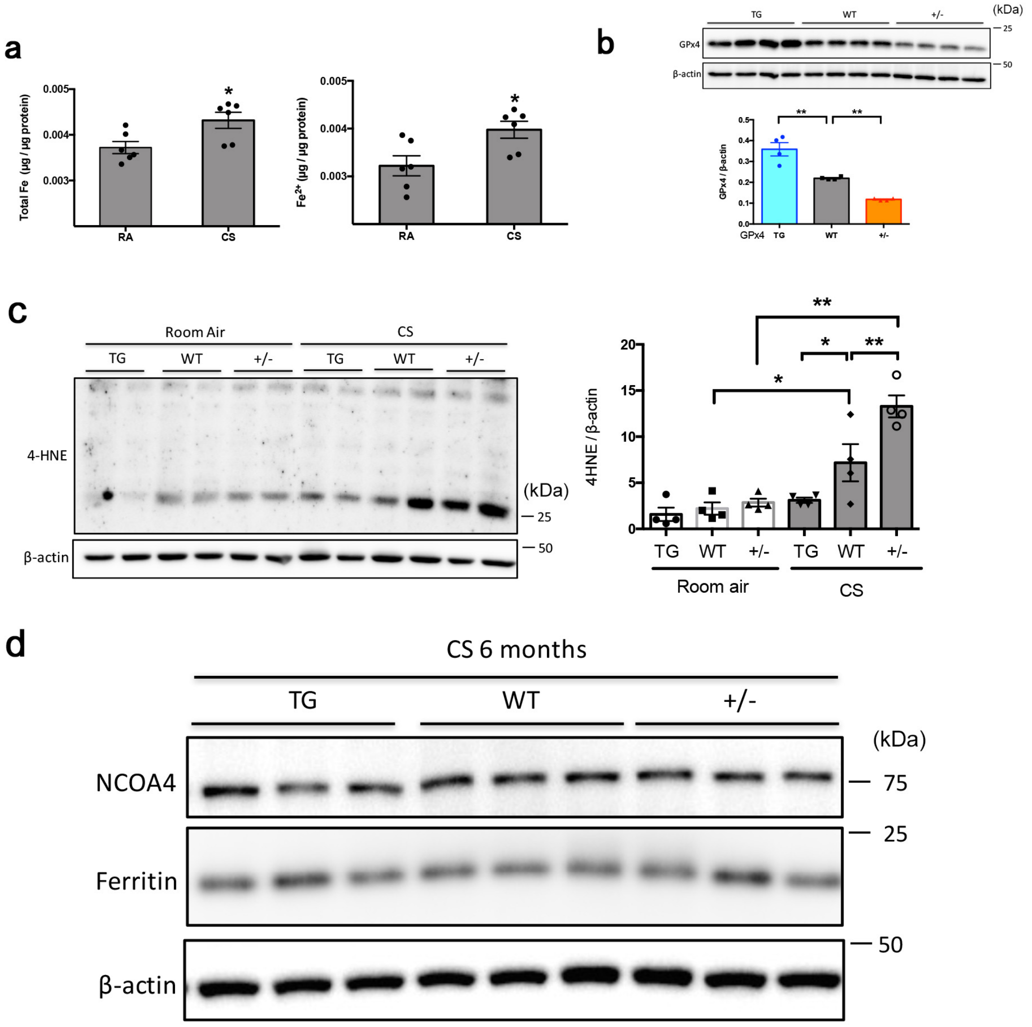

Supplementary Figure 3

a. Total and ferrous iron concentration in lung homogenates from RA or CS exposed mice .n=6 in each group. Data shown represent the mean ±SEM. *P<0.05 by student t test. **b.** Western blotting (WB) showing expression levels of GPx4 in GPx4+/-,

WT(GPx4+/+), and GPx4 TG (TG(loxP-GPx4):GPx4+/+) mice lung homogenate. n=4 in each group Data shown represent the mean ±SEM. *P<0.05, **P<0.01 by one-way ANOVA followed by Tukey's multiple comparisons test. **c.** Mice were exposed to RA or CS for 4 weeks. WB showing expression levels of 4-HNE and β-actin in $GPx4^{+/-}$, WT, and GPx4 TG (TG(loxP-GPx4):$GPx4^{+/+}$) mouse lung homogenates. n=4 in each group. Data shown represent the mean ±SEM of 4-HNE/ β-actin ratio. *P<0.05, **P<0.01 by one-way ANOVA followed by Tukey's multiple comparisons test. **d.** WB showing expression levels of NCOA4 and β-actin in $GPx4^{+/-}$, WT, and GPx4 TG (TG(loxP-GPx4):$GPx4^{+/+}$) mouse lung homogenates. Mice were exposed to CS for 6months.

Supplementary Figure 4.

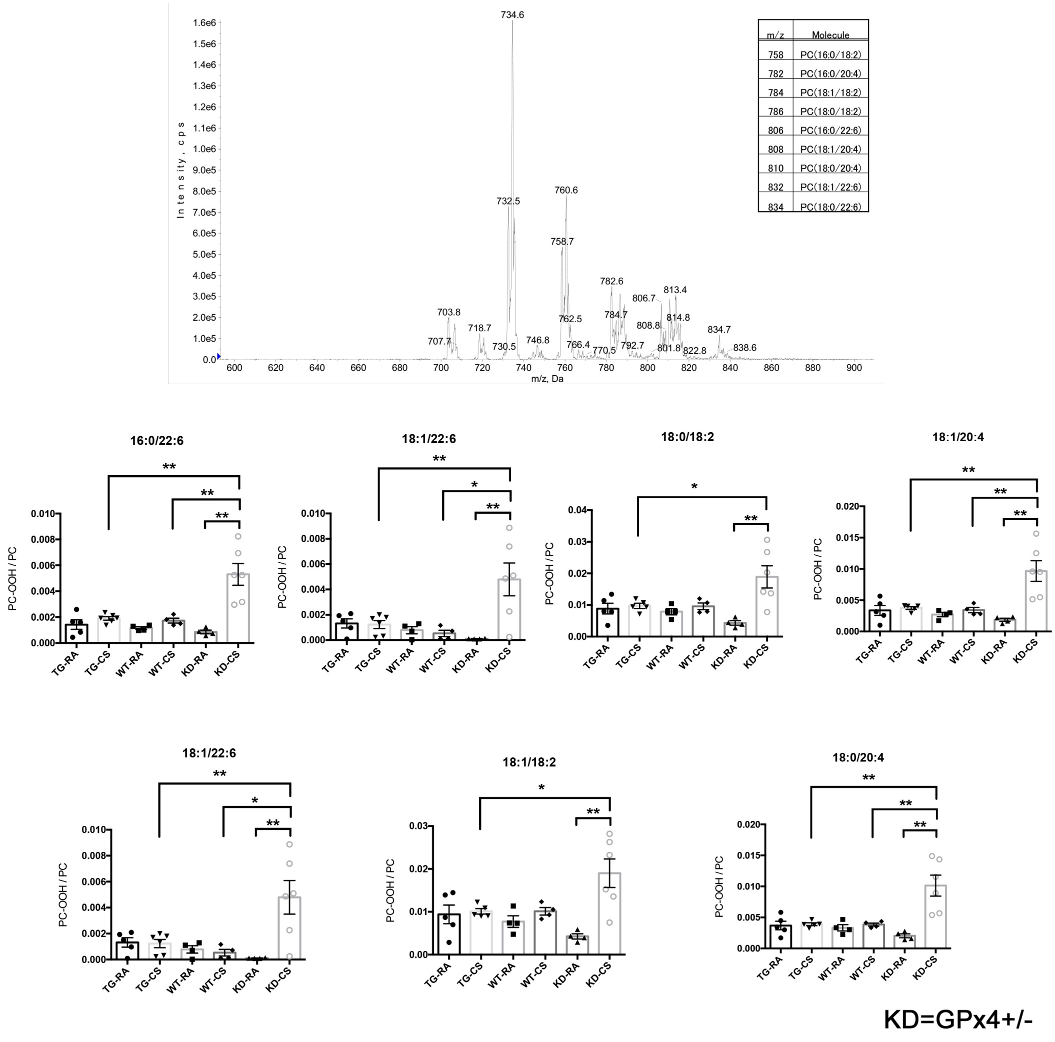

Supplementary Figure 4

LC-MS analysis of PC and their oxidation products in mouse lung homogenate. Mass spectrum of PC were shown in upper panels. PC-OOH/PC (16:0/22:6), (18:0/22:6), (18:0/18:2), (18:1/20:4), (18:1,22:6), (18:1/18:2), (18:0/20:4) in GPx4+/-, WT, and GPx4 TG (TG(loxP-GPx4):GPx4+/+) mouse lung homogenates are shown in bottom panels. Mice were exposed to Room Air (RA) or cigarette smoke (CS) for 6 months. TG RA n=5, TG CS n=6, WT RA n=4, WT CS n=4, +/- RA n=4, +/- CS n=6. Data shown represent the

mean ±SEM. *P<0.05, **P<0.01 by one-way ANOVA followed by Tukey's multiple comparisons test.

Supplementary Figure 5.

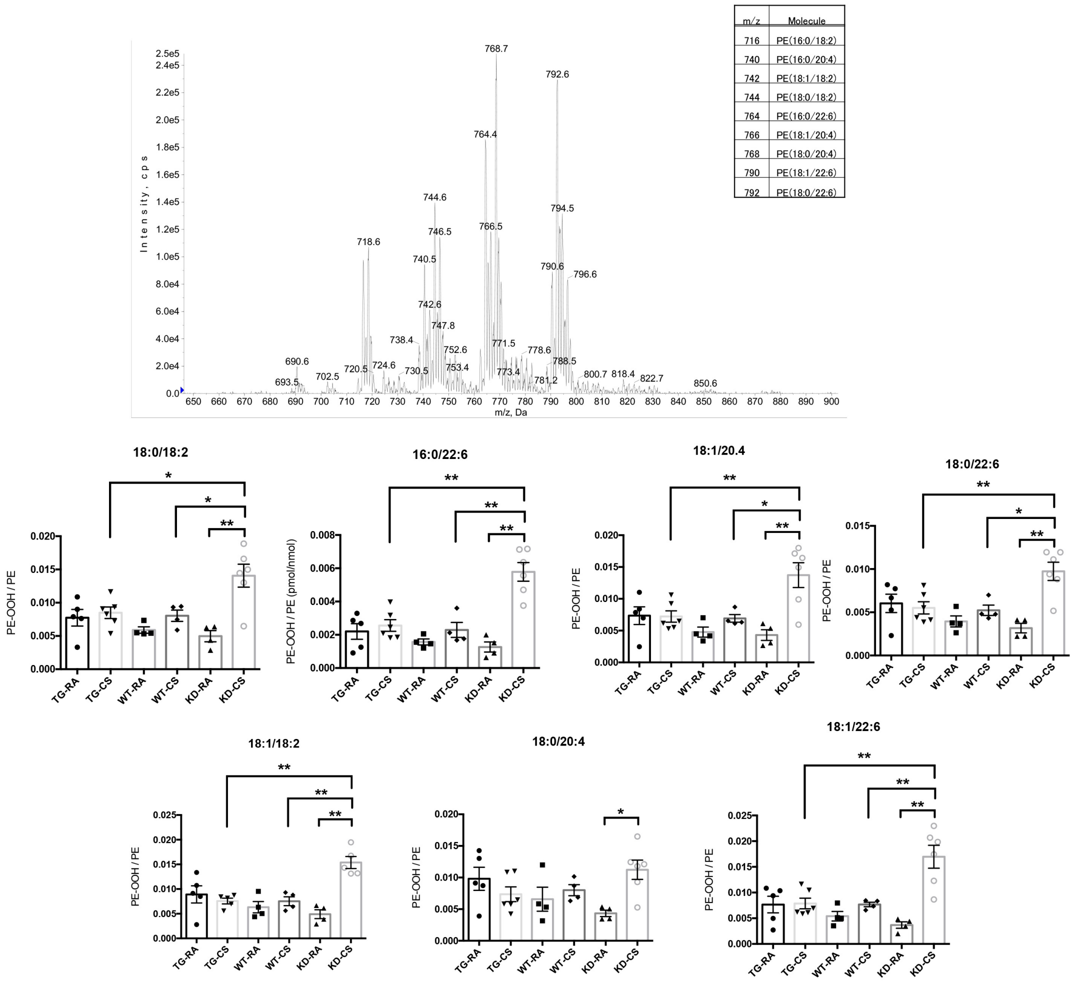

KD=GPx4+/-

Supplementary Figure 5

LC-MS analysis of PE and their oxidation products in mouse lung homogenate. Mass spectrum of neutral loss scanning of 141 Da in the positive ion mode were shown in upper panels. PE-OOH/PE (18:0/18:2), (16:0/22:6), (18:1/20:4), (18:0/22:6), (18:1/18:2), (18:0/20:4), (18:1,22:6) in GPx4+/-, WT, and GPx4 TG (TG(loxP-GPx4):GPx4+/+) mouse lung homogenates are shown in bottom panels. Mice were exposed to Room Air (RA) or cigarette smoke (CS) for 6 months. Data shown represent the mean ±SEM. TG RA n=5,

TG CS n=6, WT RA n=4,WT CS n=4,+/- RA n=4, +/- CS n=6.   *P<0.05, **P<0.01 by one-way ANOVA followed by Tukey's multiple comparisons test.

Supplementary Figure 6

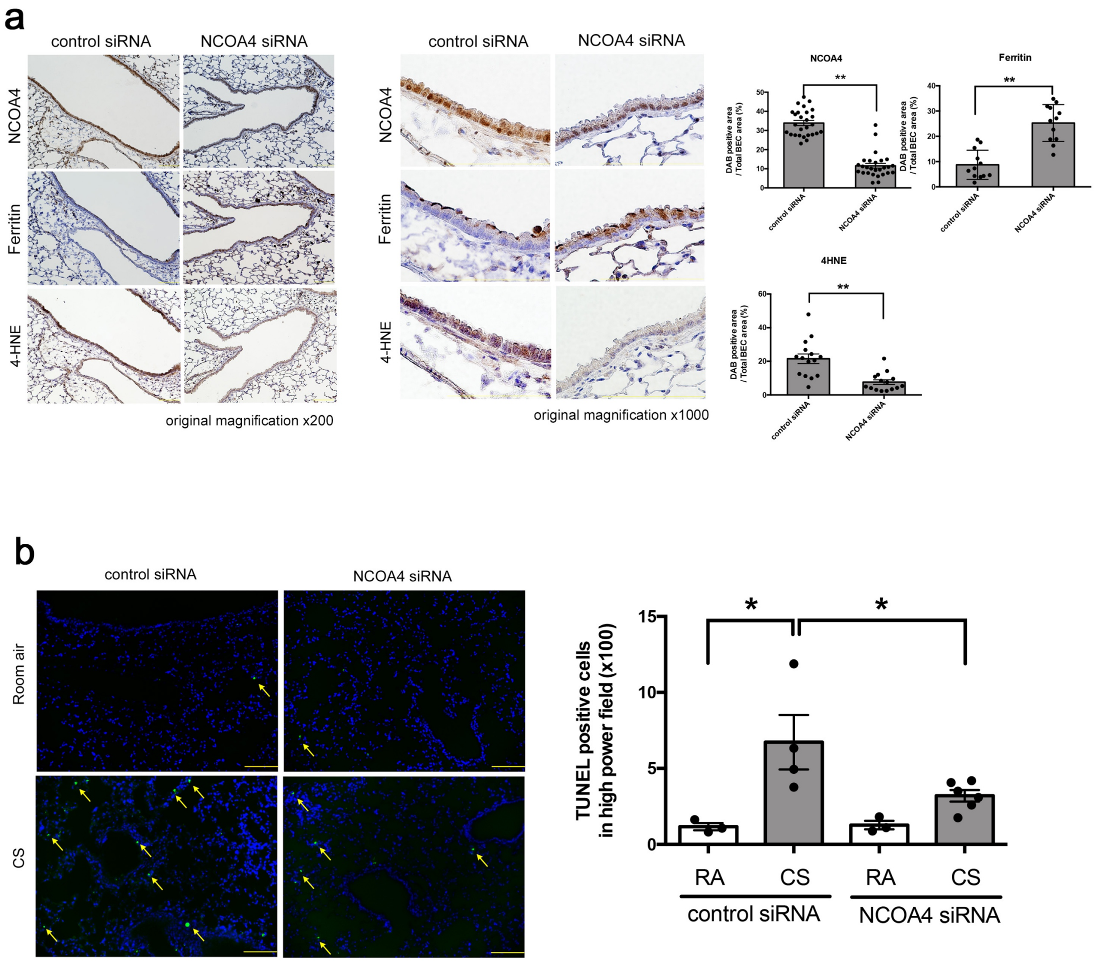

Supplementary Figure 6

WT(GPx4+/+) mice were exposed to CS for 7days. Control siRNA or NCOA4 siRNA was injected intra-tracheally on day1. **a.** Immunohistochemical staining of NCOA4 (upper panels), Ferritin (middle panels), and 4-HNE in mouse lung airway (lower panels). Original magnification x200 (left panels), x1000 (right panels). Bar=100μm. NCOA4 9 random bronchus were chosen blindly. Ferritin 4 random bronchus were chosen blindly.

4HNE 5 random bronchus were chosen blindly. 3mice/each group were analyzed. Data shown represent the mean ±SEM of NCOA4, Ferritin and 4-HNE positive area / total bronchial epithelial cells area ratio. **P<0.01 by student t test. **b.** TUNEL assay staining (green) of GPx4 in control siRNA or NCOA4siRNA injected WT(GPx4+/+) mice lung sections. Nuclei were counterstained with DAPI (blue). Data shown represent the mean ±SEM of TUNEL positive cell counts. Control siRNA RA n=3, control siRNA CS n=4, NCOA4 siRNA RA n=3, NCOA4 siRNA CS n=6 . *P<0.05, **P<0.01 by one-way ANOVA followed by Tukey’s multiple comparisons test.

Supplementary Figure 7.

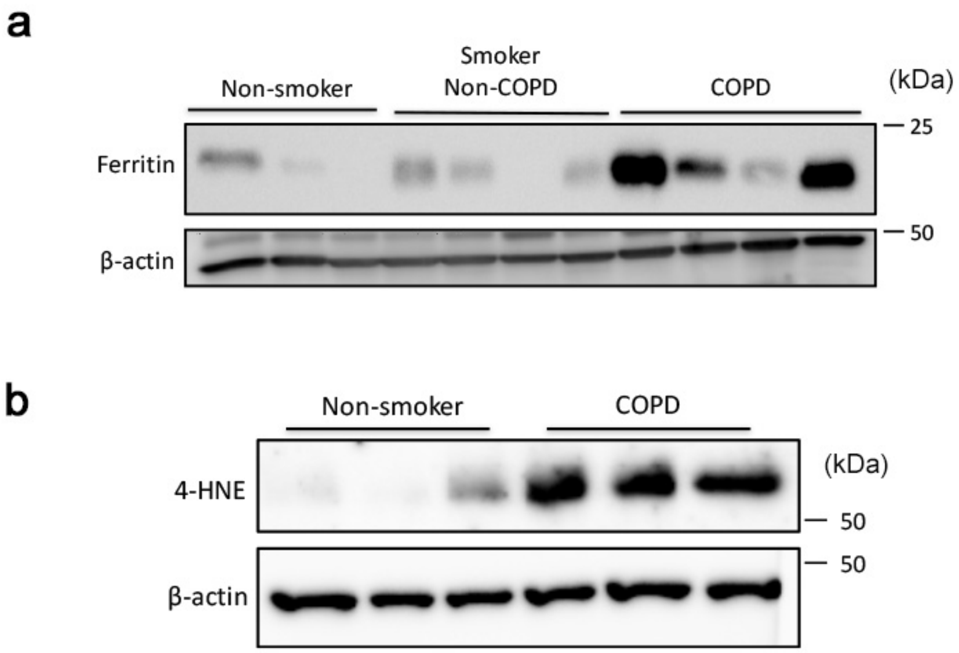

Supplementary Figure 7

a. WB using anti-ferritin and anti-β-actin of lung homogenates from non-smoker, smoker without COPD, and COPD. **b.** WB using anti-4-HNE and anti-β-actin of lung homogenates from non-smoker and COPD.

Supplementary Figure 8

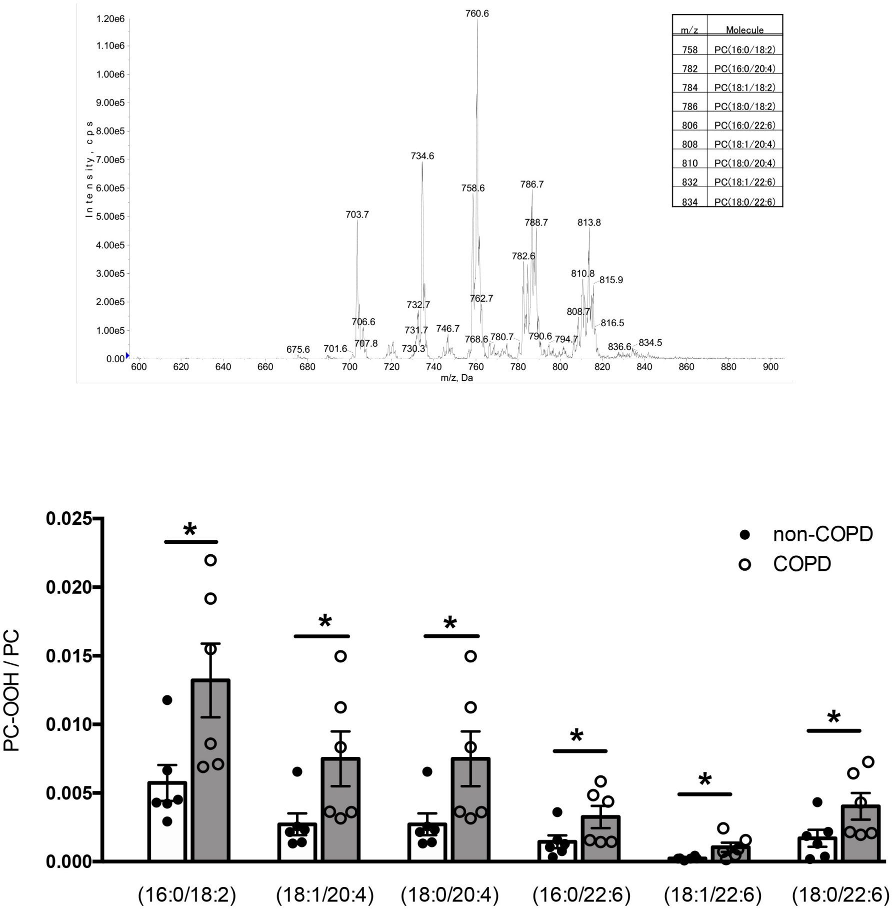

Supplement Figure 8

LC-MS analysis of PC and their oxidation products in human lung homogenates. Mass spectrum of PC were shown in upper panels. PC-OOH/PC (16:0/18:2), (18:1/20:4), (18:0/20:4), (16:0/22:6), (18:1/22:6), (18:0/22;6) in non-COPD and COPD lung homogenates are shown in bottom panels. Data shown represent the mean ±SEM of n=6 in each group. *P<0.05 by student's t test.

Supplementary Figure 9

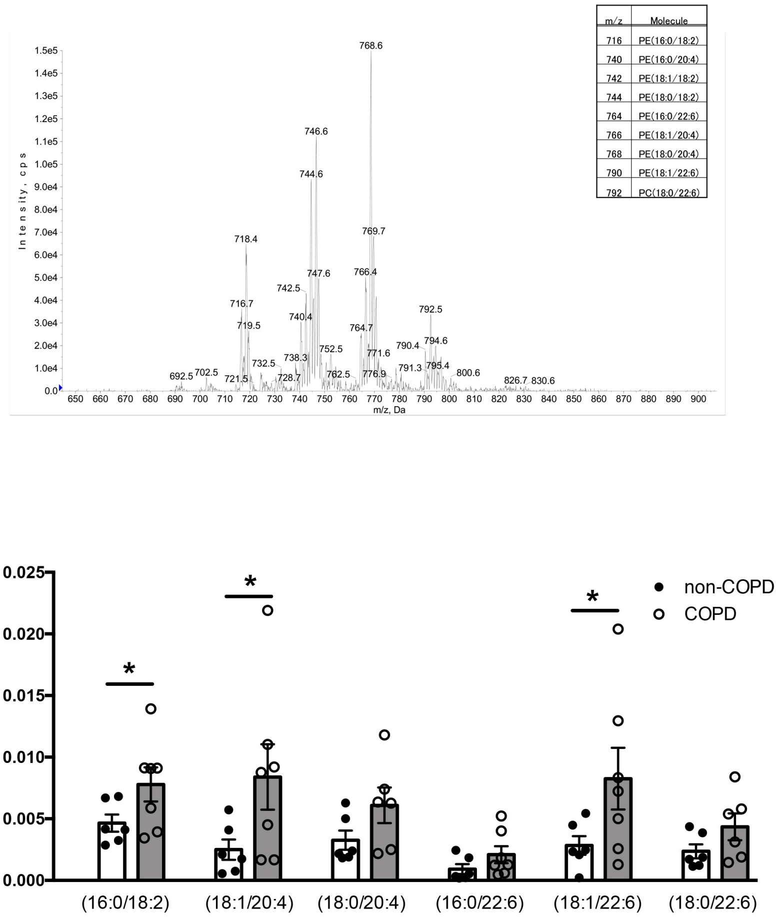

Supplementary Figure 9

LC-MS analysis of PE and their oxidation products in human lung homogenates. Mass spectrum of PE were shown in upper panels. PE-OOH/PE (16:0/18:2), (18:1/20:4), (18:0/20:4), (16:0/22:6), (18:1/22:6), (18:0/22;6) in non-COPD and COPD lung homogenate are shown in bottom panels. Data shown represent the mean ±SEM. Non COPD n=6, COPD n=7. *P<0.05 by student's t test.

Supplementary Figure 10.

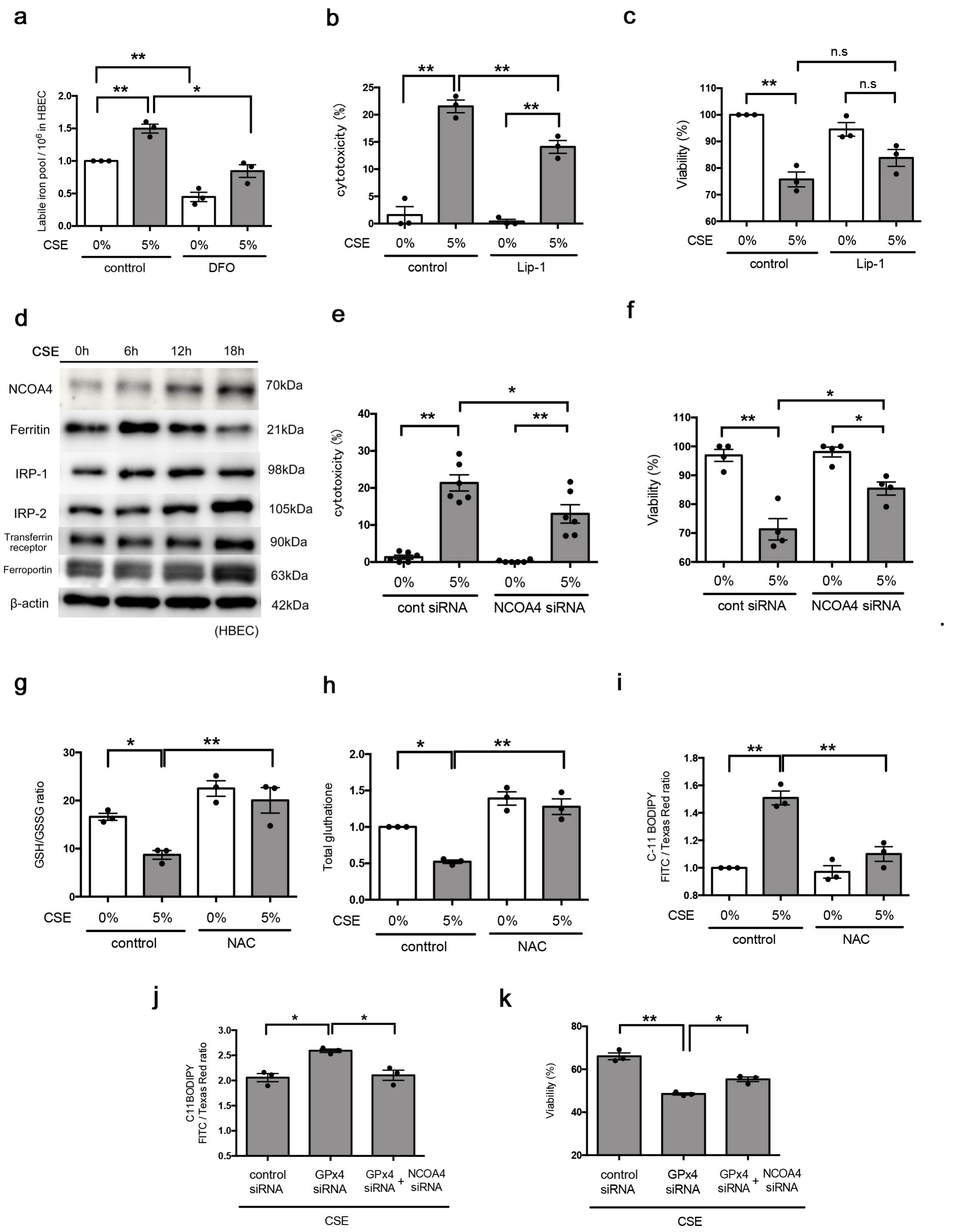

Supplementary Figure 10

a. HBECs were treated with 5% CSE for 24 hours. Labile iron pool was measured using calcein-AM method in control or 5% CSE treated HBECs. Saline or DFO (100μM) was added to HBECs 1hr before control or 5% CSE treatment. n=3 in each group. **b,c.** HBECs were treated with 5% CSE for 24 hours. Cell death was assessed by cytotoxicity using **b.** LDH assay and **c** cell viability using MTT assay. Saline or Liproxstatin-1 (Lip-1) (2μM) was added to HBECs 1h before control or 5% CSE treatment. n=3 in each group. **d.** HBECs were treated with 5% CSE for 24 hours. Time course of ferritin, NCOA4, IRP-1, IRP-2, Transferrin receptor, Ferroportin, and β-actin expression in response to 5% CSE were assessed by WB. **e.** LDH assay and **f** MTT assay are shown. HBECs were transfected with control siRNA or NCOA4 siRNA 48h before control or CSE treatment. **e.** n=6 in each group. **f.** n=4 in each group. **g,h,i,j,k.** HBECs were treated with 5% CSE for 24 hours. Saline or N-acetylcysteine (1mM) was added to HBEC 1h before control or 5% CSE treatment. **g**.GSH/GSSG ratio **h**.total glutathione levels and **i.** lipid peroxidation (BODIPY581/591 staining) were analyzed. **j,k.** HBECs were transfected with control, GPx4, or GPx4 plus NCOA4 siRNA 48h before control or CSE treatment. **j.**Lipid peroxidation (BODIPY581/591 staining). and **k.**cell viability (MTT assay) . were analyzed. **g,h,i,j,k** n=3 in each group.

Throughout, data shown represent the mean ± SEM. *P<0.05, **P<0.01 by one-way ANOVA followed by Tukey’s multiple comparisons test.

Supplementary Table 1

Antibody	Cat no.	dilution
GPx4	125066 (Abcam)	1:1000 (WB), 1:200 (IHC-P)
Ferritin Heavy Chain	65080 (Abcam)	1:1000 (WB), 1:200 (ICC)
NCOA4	86707 (Abcam)	1:1000 (WB), 1:100 (IHC-P)
ATG5	2630 (CST)	1:1000 (WB)
caspase-3	9665 (CST)	1:1000 (WB)
cleaved caspase-3	9664 (CST)	1:400 (IF-F)
4-HNE	46545 (Abcam)	1:2000 (WB), 1:300 (IHC-P)
IRP-1	20272 (CST)	1:1000 (WB)
IRP-2	37135 (CST)	1:1000 (WB)
Transferrin receptor 1 (CD71)	13113 (CST)	1:1000 (WB)
Ferroportin-1	49668 (Santa Cruz)	1:1000 (WB)
β-actin	A5316 (Sigma-Aldrich)	1:3000 (WB)

Supplementary Table 1 – Antibodies used for experiment.

WB= Western blot, IHC-P = Immunohistochemistry-Paraffin, ICC = Immunocytochemistry,

IF-F = Immunofluorescence-frozen
