# Supplementary material for: Involvement of cigarette smoke-induced epithelial cell ferroptosis in COPD pathogenesis
Source: Nat Commun. 2019 Jul 17;10:3145. doi: 10.1038/s41467-019-10991-7 (PMC6637122; doi:10.1038/s41467-019-10991-7)
Supplement: Supplementary file 3 — Reporting summary [file 41467_2019_10991_MOESM3_ESM.pdf]

natureresearch

Corresponding author(s): Shunsuke Minagawa

Last updated by author(s): May 4, 2019

Reporting Summary

Nature Research wishes to improve the reproducibility of the work that we publish. This form provides structure for consistency and transparency in reporting. For further information on Nature Research policies, see Authors & Referees and the Editorial Policy Checklist.

Statistics

For all statistical analyses, confirm that the following items are present in the figure legend, table legend, main text, or Methods section.

n/a	Confirmed	
☐	☒	The exact sample size (n) for each experimental group/condition, given as a discrete number and unit of measurement
☐	☒	A statement on whether measurements were taken from distinct samples or whether the same sample was measured repeatedly
☐	☒	The statistical test(s) used AND whether they are one- or two-sided<br>*Only common tests should be described solely by name; describe more complex techniques in the Methods section.*
☐	☒	A description of all covariates tested
☐	☒	A description of any assumptions or corrections, such as tests of normality and adjustment for multiple comparisons
☐	☒	A full description of the statistical parameters including central tendency (e.g. means) or other basic estimates (e.g. regression coefficient) AND variation (e.g. standard deviation) or associated estimates of uncertainty (e.g. confidence intervals)
☐	☒	For null hypothesis testing, the test statistic (e.g. F, t, r) with confidence intervals, effect sizes, degrees of freedom and P value noted<br>*Give P values as exact values whenever suitable.*
☒	☐	For Bayesian analysis, information on the choice of priors and Markov chain Monte Carlo settings
☒	☐	For hierarchical and complex designs, identification of the appropriate level for tests and full reporting of outcomes
☒	☐	Estimates of effect sizes (e.g. Cohen's d, Pearson's r), indicating how they were calculated

Our web collection on statistics for biologists contains articles on many of the points above.

Software and code

Policy information about availability of computer code

Data collection	no software used
Data analysis	Prism v.6 (GraphPad Software, Inc., San Diego,CA), Image J

For manuscripts utilizing custom algorithms or software that are central to the research but not yet described in published literature, software must be made available to editors/reviewers. We strongly encourage code deposition in a community repository (e.g. GitHub). See the Nature Research guidelines for submitting code & software for further information.

Data

Policy information about availability of data

All manuscripts must include a data availability statement. This statement should provide the following information, where applicable:

- Accession codes, unique identifiers, or web links for publicly available datasets
- A list of figures that have associated raw data
- A description of any restrictions on data availability

The data that support the findings of this study are available from corresponding author upon reasonable request.

Field-specific reporting

Please select the one below that is the best fit for your research. If you are not sure, read the appropriate sections before making your selection.

☒ Life sciences ☐ Behavioural & social sciences ☐ Ecological, evolutionary & environmental sciences

For a reference copy of the document with all sections, see nature.com/documents/nr-reporting-summary-flat.pdf

Life sciences study design

All studies must disclose on these points even when the disclosure is negative.

Sample size	In each experiment, at least three samples were used for statical evaluation . Sample size were not calculated because this study is not a clinical trials.
Data exclusions	Outliers were determined by Grubb's test and excluded (GraphPad Software).
Replication	At least three independent experiments were performed in each experiment.
Randomization	All samples were anonymized by labeling with an identifying code.
Blinding	All histpathologic evaluation was assessed by blinded investigator.

Reporting for specific materials, systems and methods

We require information from authors about some types of materials, experimental systems and methods used in many studies. Here, indicate whether each material, system or method listed is relevant to your study. If you are not sure if a list item applies to your research, read the appropriate section before selecting a response.

Materials & experimental systems

n/a	Involved in the study
☐	☒ Antibodies
☐	☒ Eukaryotic cell lines
☒	☐ Palaeontology
☐	☒ Animals and other organisms
☐	☒ Human research participants
☒	☐ Clinical data

Methods

n/a	Involved in the study
☒	☐ ChIP-seq
☒	☐ Flow cytometry
☒	☐ MRI-based neuroimaging

Antibodies

Antibodies used	rabbit anti-Ferritin Heavy Chain (Abcam, 65080), rabbit anti-NCOA4 (Abcam, 86707), rabbit anti-NCOA4(Thermo Fischer Scientific,PA5-36391) rabbit anti-ATG5 (Cell Signaling Technology, 2630), rabbit anti-caspase-3 (Cell Signaling Technology, 9665), rabbit anti-cleaved caspase-3 (Cell Signaling Technology, 9664), rabbit anti-GPx4 (Abcam, 125066), rabbit anti-4-HNE (Abcam, 46545), rabbit anti-IRP-1(Cell signaling, 20272), rabbit anti-IRP-2(Cell signaling, 37135), rabbit anti-Transferrin receptor (Cell signaling, 13113), goat anti-Ferroportin (Santa cruz,sc-49668), and mouse anti-β-actin (Sigma-Aldrich, A5316).
Validation	All antibodies validation statement is shown in manufacturer's website.

Eukaryotic cell lines

Policy information about cell lines

Cell line source(s)	Bronchial epithelial cell line BEAS-2B (ATCC,Manassas,VA),<br>The alveolar type II cell-like epithelial cell line A-549 cells (ATCC,Manassas,VA)
Authentication	None of cell lines use were authenticated.
Mycoplasma contamination	Cell lines were not tested for mycoplasma contamination.
Commonly misidentified lines<br>(See ICLAC register)	No misidentified lines.

Animals and other organisms

Policy information about studies involving animals; ARRIVE guidelines recommended for reporting animal research

Laboratory animals	In each experiment, age and sex are matched in each group. 6-8 week old mice were used in all experiments. C57BL/6 mice were obtained from CLEA Japan, Inc for ICP-MS experiment. Heterozygous GPx4-deficient mice (GPx4+/-) and GPx4 Transgenic mice (TG (loxP-GPx4):GPx4+/+) on mixed back ground (TT2, ICR, BDF1 strains) were provided by Prof. Hirotaka Imai, Kitasato university9. We used GPx4+/+ on the same background as controls . GPx4+/+mice were also used for iron accumulation and NCOA4 expression experiment (Fig. 3a,c,d)

Wild animals	This study did not involve wild animals.
Field-collected samples	This study did not involve field-collected samples.
Ethics oversight	Animal experiments were approved by the committee on Animal Care at th Jikei University and performed in accordance with institutional guidelines.

Note that full information on the approval of the study protocol must also be provided in the manuscript.

Human research participants

Policy information about studies involving human research participants

Population characteristics	Patients who took surgical operation (lobectomy) for lung cancer and provided informed consent to us. Older than 20years old.
Recruitment	N/A
Ethics oversight	ethical committee of Jikei University School of Medicene (#20-153 (5443))

Note that full information on the approval of the study protocol must also be provided in the manuscript.
